# Anthracycline-induced arrhythmias in breast cancer therapy: A meta-analysis of single-arm trials

**DOI:** 10.1371/journal.pone.0303208

**Published:** 2024-05-23

**Authors:** Tao Ran, Jinyao Chen, Qiurui She, Yi Mu, Min Zhang, Min Mao, Zhong Zuo, Juan Li

**Affiliations:** 1 Department of Cardiology, The First Affiliated Hospital of Chongqing Medical University, Chongqing, China; 2 School of the First Clinical Medical College, Chongqing Medical University, Chongqing, China; 3 School of Public Health and Management, Chongqing Medical University, Chongqing, China; 4 Department of Academic Affairs, The First Affiliated Hospital of Chongqing Medical University, Chongqing, China; Dartmouth Health, UNITED STATES

## Abstract

**Introduction:**

As of 2020, breast cancer has emerged as the predominant cause of cancer incidence globally. Anthracycline-based chemotherapy serves as a crucial element in the treatment regimen for breast cancer. However, these anthracycline-based drugs are associated with cardiac toxicity. This study represents the first clinical quantitative analysis aimed at accurately determining the incidences of arrhythmia and abnormal electrocardiogram (ECG) changes, thereby providing valuable data to bolster clinical drug usage and monitoring.

**Methods:**

A systematic search was conducted across multiple databases including CNKI, VIP, Wanfang, PubMed, Embase, Web of Science, and the Cochrane Library. The incidence of combined arrhythmias in breast cancer patients and the associated heterogeneity were calculated using either a random effect model or a fixed effect model. Statistical analysis was performed using STATA16.

**Results:**

The study encompassed a total of 37 articles, which included 5705 breast cancer patients undergoing anthracycline treatment. Among these patients, 2257 developed arrhythmias. The meta-analysis revealed that the incidence of anthracycline-associated arrhythmias and abnormal ECG changes in breast cancer patients was 0.41 (0.37, 0.44). Subgroup analysis indicated that the incidence of ST-T segment change was 0.19 (0.15, 0.23), the incidence of conduction block was 0.04 (0.02, 0.05), the incidence of premature beats was 0.09 (0.07, 0.11), and the incidence of atrial fibrillation was 0.04 (0.00, 0.12). Additional results are presented in Table 3.

**Conclusion:**

This pioneering study accurately assesses the incidence of arrhythmias in breast cancer patients treated with anthracyclines. The findings provide clinicians with valuable insights into understanding and managing the cardiac toxicity associated with such treatment. Moreover, this study lays the foundation for future research exploring the mechanisms underlying these arrhythmias and potential preventative strategies.

## Introduction

By 2020, breast cancer had surpassed lung cancer to become the predominant cause of cancer incidence globally and the fifth leading cause of cancer death [1]. From the 1990s to the early 21st century, an average of 1.15 million people were diagnosed with breast cancer annually, with 410,000 succumbing to the disease each year [2]. The primary treatment modality for breast cancer is systemic comprehensive treatment, primarily based on surgery. Studies have indicated that adjuvant chemotherapy can reduce the 10-year recurrence risk of breast cancer by 23.5% and the 10-year mortality risk by 15%, thereby prolonging patient survival and reducing recurrence [3]. Consequently, most adenocarcinoma patients, barring a few with a low risk of recurrence and intolerance to chemotherapy, require adjuvant chemotherapy post-surgery, with anthracycline-containing regimens playing a pivotal role in breast cancer therapy [4].

Anthracyclines, which include daunorubicin (DNR), adriamycin (ADM), doxorubicin, pirarubicin, mitoxantrone (MIT), and carubicin, are landmark drugs in the field of medical oncology. They are extensively used in the treatment of hematological malignancies and solid tumors, including acute leukemia, lymphoma, breast cancer, ovarian cancer, gastric cancer, and soft tissue sarcoma [5]. Despite their potent anticancer effects, the clinical use of anthracyclines is limited by their toxicity profile, which includes alopecia, bone marrow suppression, and cardiotoxicity, the latter being the most severe [6]. Cardiotoxicity induced by anthracyclines can manifest as arrhythmia, intracardiac conduction disorder, pericarditis, heart failure, cardiomyopathy, and can be categorized into acute, chronic, and delayed cardiotoxicity. Studies have demonstrated that the use of anthracyclines in the treatment of breast cancer patients can result in conduction block, palpitations, QT prolongation, ventricular arrhythmias, supraventricular arrhythmias, and other types of electrocardiogram (ECG) changes and arrhythmias [6–8]. Anthracycline-induced arrhythmias occur in a dose-dependent manner. Taking adriamycin as an example, studies have shown that when the cumulative dose of adriamycin reaches 300 mg/m2, the incidence of arrhythmia is 0.34, while an incidence of arrhythmia of 0.53 was found for a cumulative doxorubicin dose of 450 mg/m^2^ [9].

Since the cardiotoxicity of anthracycline was first reported by Lefrak in 1973, numerous studies have reported on the incidence and clinical characteristics of anthracycline cardiotoxicity in breast cancer patients. However, the sample size of each independent study is relatively small, and there are substantial discrepancies among the results of each study [10–13]. Furthermore, there are few reviews on the toxicity and side effects of anthracyclines in the treatment of breast cancer-induced arrhythmias, and quantitative analysis is lacking [14,15].

To enhance the accuracy of the statistics and the credibility of the results, this quantitative analysis was undertaken to accurately assess the incidences of overall arrhythmia and specific subtypes of arrhythmia and electrocardiogram (ECG) changes. This will help to better define the side effects of anthracycline-based drugs and provide supporting data for clinical drug use and drug monitoring.

## Material and methods

This meta-analysis was conducted in accordance with the Meta-analyses of Observational Studies in Epidemiological (MOOSE) guidelines. And the study has been registered in the international prospective register of systematic reviews(PROSPERO) Registration No: CRD42022321213. The present study was granted an exemption from requiring ethics approval by Chongqing Medical University because it did not involve human subjects.

### Literature retrieval strategy

A comprehensive search was performed across three Chinese databases (CNKI, VIP, and Wanfang) and four English databases (PubMed, Embase, Web of Science, and Cochrane Library). The specific search strategy is presented in S1 Table. And the retrieval time was from the establishment of the database to March 1, 2023.Two independent researchers conducted the literature search and screening. In case of discrepancies, a consensus was reached through active discussion with a third party.

### Inclusion and exclusion criteria

#### Inclusion criteria

Only clinical studies with a sample size of ≥20 were included in the analysis without limiting the type of study, provided they met the following criteria: (1) Studies that included patients diagnosed with breast cancer via histopathology. (2) Studies that included patients who had received anthracyclines (including daunorubicin, doxorubicin/epirubicin/, mitoxantrone, and carubicin) alone or anthracycline-based chemotherapy. (3) Studies that reported the incidence of arrhythmia events and indicators of ECG abnormalities. (4) Studies that administer cardioprotective drugs and include their control groups for analysis.

#### Exclusion criteria

(1) Reviews, case reports, conference papers, and animal or in vitro studies.(2) Identical or duplicate studies (the latest research results were considered).(3) Studies where the full text was unavailable.(4) Studies lacking dose or duration data.(5) Studies not specifying the number of arrhythmias.

### Control of literature collection bias

Two researchers independently conducted the literature retrieval. A retrieval strategy was used to organize and screen studies and eliminate duplicate documents. The remaining studies were screened by reading the title and abstract. If the study did not meet the inclusion criteria, it underwent preliminary screening. If the study still did not meet the inclusion criteria, the full text of the study was read again. Ultimately, the studies that met the inclusion criteria were included in the study. In case of disagreement, a third reviewer was consulted, and the final decision on inclusion was made through collective discussion with field experts and scholars.

### Risk assessment of literature bias

The quality of each study was evaluated using the methodological evaluation indices for nonrandomized controlled experiments proposed by Slim et al. in 2007. There were 12 evaluation indices, each scored with 0–2 points. For the first eight studies without a control group, the maximum score was 16. For the last four and first eight studies that included a control group, the highest score was 24 points. A score of 0 indicated no reporting; a score of 1 indicated reporting of insufficient information; 2 indicated reporting of sufficient information; < 12 indicated low-quality literature; and ≥12 indicated high-quality literature.

### Data extraction

Two evaluators independently extracted and processed the data from the included literature and uploaded the data according to their preferred formats. Upon completion, they cross-checked the data. In case of any inconsistency, a third evaluator was included, and the final results were determined through collective discussion. The extracted data included the first author’s name, year of publication, study type, number of patients, patient age, treatment regimen, treatment dose, administration mode, treatment duration, number of patients lost to follow-up, incidence of arrhythmia, and abnormal ECG changes.

### Statistical analysis

Statistical analysis was performed using STATA16 software, with the incidence of arrhythmia serving as the primary effect index. Given the number of patients and extreme differences in proportions in some studies, a 95% confidence interval (CI) was calculated using the Wilson score. As the incidence data were not normally distributed, the incidence rates reported in all studies were calculated by logit transformation after combination. Cochran’s Q-test and *I*^2^ statistical data were used to evaluate the heterogeneity between studies. A fixed-effect model was employed when P>0.1 and *I*^2^<50%; otherwise, a random effects model was used. Publication bias was evaluated using funnel plots and Egger’s and Begg’s tests. In addition, we conducted subgroup analyses based on publication year, sample size, dose and duration of administration, treatment plan, and the timing of cardiac toxicity, ECG changes, and arrhythmia. Sensitivity analysis was then performed to test the stability of the meta-analysis results. Tests of heterogeneity and bias were one-tailed, and a p value less than 0.10 was considered significant. A two-sided p value of 0.05 was considered statistically significant..

## Results

### Literature retrieval results

**Fig 1:**
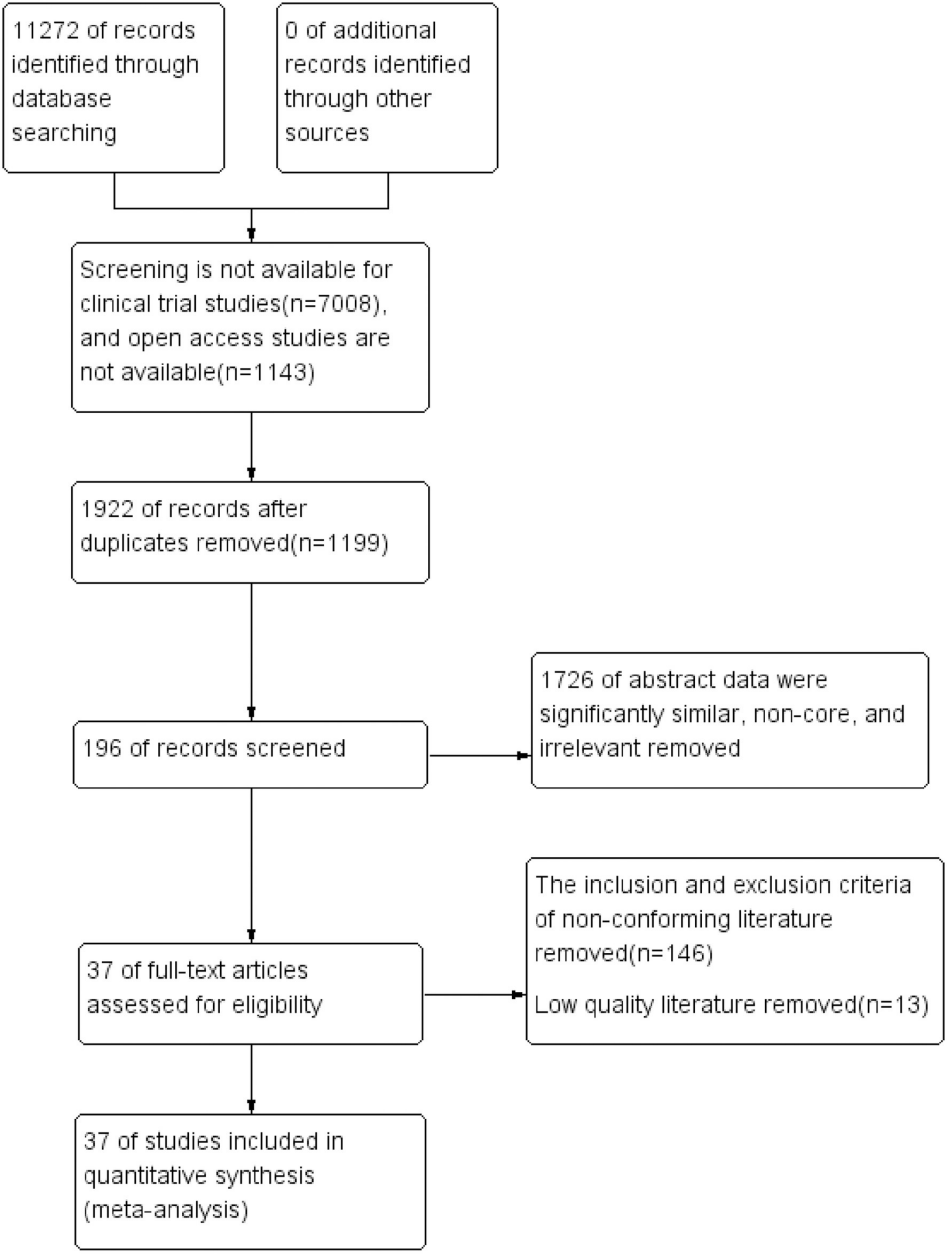
Study flow diagram.

In accordance with the retrieval strategy, a total of 11,272 articles were retrieved. Following a rigorous quality evaluation and reference check, 37 studies were derived from references. The document selection flow chart is depicted in Fig 1.

### Basic characteristics of the included studies

The analysis included a total of 37 studies. The total sample size was 5706, including one patient lost to follow-up, resulting in an actual sample size of 5705. Given that 12 studies divided the patients into multiple study groups based on various factors (drug, dose, duration, etc.), a total of 46 treatment groups were reviewed. Of these, 13 were treated with an anthracycline alone, most commonly doxorubicin, and 33 were treated with combination chemotherapy, such as CAF, TA or TE, with CAF being the most common. Eleven of the 46 studies assessed agents protecting against chemotherapy-associated arrhythmias, for which only control data (placebo rather than a cardioprotective agent) were used. Simultaneously, cardiac toxicity occurred during chemotherapy period(first cycle of chemotherapy—time until 3 weeks after the last cycle of chemotherapy) in 42 studies, while in 4 studies, cardiac toxicity occurred in post-chemotherapy(time after 3 weeks following the last cycle of chemotherapy). The basic characteristics of the included studies are presented in Table 1.

**Table 1:** The basic characteristics of the included studies.

Study	Year	Language	Total Patients	No. Event	Changes in ECG	Subtype of Events	Treatmen-t plan	Treatment cycle	Dosage(mg/m^2^)	Administratio-n mode	The timing of cardiac toxicity	MINO-RS score
He F	2017	Chinese	213	72	T wave change(40)ST-T segment change(11)	Conduction block(5)Sinus tachycardia(13)Sinus bradycardia(6)Sinus arrhythmia(6)Ventricular premature beat(1)	ADR	1-6cycle	/	/	Chemotherapy period	12
Zhang KK	2014	Chinese	78	45	ST segment change(15) QT interval change(4)	Atrial premature beat(8) Ventricular premature beat(6) Sinus arrhythmia(12)	TA	4cycle	200	/	Chemotherapy period	14
Yi SY	2008	Chinese	30	9	ST-T segment change(4)QRS low voltage(1)	Sinus tachycardia(2)Premature beat(2)	PA	4cycle	200	iv	Chemotherapy period	13
Zhang MM	2018	Chinese	291	133	ST segment change(48)	Atrial premature contraction(41)Ventricular premature contraction(42)	AC/EC	6-8cycle	240–320	/	Chemotherapy period	15
Pan Y	2008	Chinese	59	21	ST-T segment change(5)QRS low voltage(2)	Ventricular premature contraction(1)Third degree atrioventricular block(1)Sinus tachycardia(2)	EPI	6M-5Y	32–478.6	iv	Chemotherapy period	12
Zhang SJ	2011	Chinese	50	12	ST-T segment change(3)Low voltage(1)	Sinus tachycardia(3)Ventricular premature beat(4)Atrial premature beat(1)	FAC	6cycle	300	/	Chemotherapy period	12
Feng LF	2014	Chinese	40	17	ST-T segment change(10)	Atrial premature contraction(2)Ventricular premature contraction(1)Atrioventricular block(1)Sinus bradycardia/Sinus tachycardia(3)	TAC	6cycle	300	/	Post-chemotherapy	17
Wang F	2016	Chinese	20	7	ST segment depression(1)T wave change(2)	Premature beat(2)Sinus tachycardia(1)First degree atrioventricular block(1)	TE	6cycle	450	p.o.	Chemotherapy period	17
Ma L	2000	Chinese	83	26	Low voltage(6)ST-T segment change(5)	Sinus tachycardia(9)Atrial premature beat(2)Ventricular premature beat(3)RBBB(1)	ADR	3-10cycle	120–400	iv	Chemotherapy period	12
Ding SQ	2014	Chinese	26	16	ST-T segment change(8)P wave change(3)	Sinus bradycardia/Sinus tachycardia(4)Premature beat(1)	AC/EC	4cycle	450–550	/	Chemotherapy period	12
Ding SQ	2014	Chinese	26	12	ST-T segment change(8)P wave change(1)	Sinus bradycardia/Sinus tachycardia(4)Premature beat(1)	AC/EC	6cycle	450–550		Chemotherapy period	12
He C	2012	Chinese	207	123	ST-T segment change(22)T wave change(17)QRS low voltage(26)QT interval change(32)	Premature beat(16)	FAC/FEC/AC	6cycle	/	/	Chemotherapy period	18
Meng KX	2015	Chinese	35	25	ST-T segment change(8)QT interval change(2)	Sinus tachycardia(2),Sinus bradycardia(1)Atrial premature contraction(10),Ventricular premature contraction(2)	EC-T	4cycle	360	iv	Chemotherapy period	18
Qi CC	2017	Chinese	123	73	ST-T segment change(30)T wave change(13)P wave change(14)Electric axis deflection(8)	Sinus tachycardia(7)Conduction block(11)	AC-T/CAF-T	6cycle	300	iv	Chemotherapy period	14
Wang ZH	2017	Chinese	78	19	ST-T segment change(9)	Atrioventricular block(3)Sinus bradycardia(2) Ventricular premature contraction(1) Atrial premature contraction(4)	AC-T	8cycle	400	/	Chemotherapy period	18
Xiong P	2006	Chinese	43	24	QT-QTc change(12)QRS low voltage(4) ST-T segment change(1)	Sinus tachycardia(2)Atrial premature beat(2)Ventricular premature beat(3)	CAF	10cycle	500	iv	Chemotherapy period	14
Xu GQ	2017	Chinese	42	17	ST-T segment change(3) QT interval change(2)	Atrial/Ventricular premature beat(9) Atrial fibrillation(7) Sinus tachycardia(12)	TEC	6cycle	420	p.o.	Chemotherapy period	17
Lv Y	2021	Chinese	191	62	/	/	AC/AC-T	1cycle	/	/	Chemotherapy period	14
Lv Y	2021	Chinese	191	76	ST-T segment change(35)	Sinus bradycardia/Sinus tachycardia(28)Atrial premature beat/Ventricular premature beat(7)Sinus arrhythmia(6)	ADR	4cycle	/	/	Chemotherapy period	14
Feng YY	2014	Chinese	232	47	ST-T segment change(15)QRS low voltage(2)	Sinus arrhythmia(17)Sinus tachycardia(12)Sinus bradycardia(5)Atrial premature beat(5)Ventricular premature beat(4)First degree atrioventricular block(2)Atrial fibrillation(1)Supraventricular tachycardia(1)	ADR	4-8cycle	240–800	iv	Chemotherapy period	13
Yang ZJ	2010	Chinese	272	88	ST-T segment change(18)QRS Low voltage(2)	First degree atrioventricular block(2)Sinus tachycardia(22)Sinus bradycardia(20)Ventricular premature beat(15)Atrial fibrillation(9)	CAF	6cycle	/	/	Chemotherapy period	12
Yang M	2018	Chinese	266	104	Short PR interval(3)ST-T segment change(17)T wave change(52)	Sinus tachycardia(27)Sinus bradycardia(9)Atrial premature contraction(8)Ventricular premature contraction(13)Atrioventricular block(1)Bundle branch block(6)	FAC/FEC/TAC/AC/EC	4cycle	/	/	Chemotherapy period	14
Cheng Y	2017	Chinese	253	92	QTc change(31)T wave change(25)ST segment change(16)Abnormal Q wave(2)	Atrioventricular block(2)RBBB(1)Borderline premature beat(1)Sinus tachycardia(10)Sinus bradycardia(9)Atrial premature contraction(7)Ventricular premature contraction(5)	AC/AC-T/TA/CAF	/	276	/	Chemotherapy period	12
Yang XL	2008	Chinese	436	155	ST-T segment change(58)QT interval change(24)QRS low voltage(16)	Sinus tachycardia(18)Atrial premature beat(22)Ventricular premature beat(15)Atrioventricular block(2)	CAF	6cycle	480–540	/	Chemotherapy period	19
Ren JL	2020	Chinese	50	22	ST-T segment change(10)	Atrial/Ventricular premature contraction(6)Sinus bradycardia/Sinus tachycardia(3)Atrioventricular block(3)	ADR	4cycle	/	/	Chemotherapy period	18
Wu YT	2020	Chinese	276	110	T wave change(57)ST segment change(38)	Sinus arrhythmia(27)Supraventricular tachycardia/Sinus tachycardia(10)	ADR	/	/	/	Chemotherapy period	13
Zhao L	2019	Chinese	58	21	ST-T segment change(8)	Ventricular premature contraction(1)Atrial premature contraction(4)Atrioventricular block(2)Sinus tachycardia or bradycardia(6)	CAF	6cycle	450	iv	Post-chemotherapy	18
Guan WF	2021	Chinese	107	53	ST-T segment change(8)QRS low voltage(6)	Sinus tachycardia(13)Sinus bradycardia(6)Ventricular premature contraction(5)Atrial premature contraction(6)Atrioventricular block(3)Bundle branch block(4)Supraventricular tachycardia(2)	ADR	4-6cycle	300–400	/	Chemotherapy period	13
Guan WF	2021	Chinese	123	40	ST-T segment change(7)QRS low voltage(4)	Sinus tachycardia(8)Sinus bradycardia(6)Ventricular premature contraction(4)Atrial premature contraction(5)Atrioventricular block(2)Bundle branch block(4)	ADR	4-6cycle	200–300	/	Post-chemotherapy	13
Feng BH	2019	Chinese	93	32	ST-T segment change(5)QRS low voltage(3)QT-QTc change(2)	Sinus tachycardia(7)Sinus bradycardia(4)Ventricular premature beat(1)Atrial premature beat(6)Bundle branch block(4)	CAF	6cycle	300	iv	Chemotherapy period	15
Feng BH	2019	Chinese	92	49	ST-T segment change(8)QRS low voltage(5)QT-QTc change(4)	Sinus tachycardia(11)Sinus bradycardia(5)Ventricular premature beat(3)Atrial premature beat(8)Supraventricular tachycardia(1)Bundle branch block(4)	CAF	6cycle	450	iv	Chemotherapy period	15
Dong XJ	2020	Chinese	279	160	QTc change(78)ST-T segment change(22)Chest lead low voltage(7)Reverse clock transposition(7)Clockwise transposition(3)Limb lead low voltage(3)Left ventricular high voltage(2)	Sinus bradycardia(18)First degree atrioventricular block(8)Ventricular premature beat(5)Sinus arrhythmia(4)Atrial premature beat(3)	ADR	4cycle	120–360	iv	Chemotherapy period	14
Chen MC	2020	Chinese	40	21	/	Frequent ventricular premature beats(20)Paired ventricular premature beats(21)Short array ventricular tachycardia(21)	THP	4cycle	600	iv	Chemotherapy period	17
Chen MC	2020	Chinese	40	27	/	Frequent ventricular premature beats(26)Paired ventricular premature beats(27)Short array ventricular tachycardia(26)	THP	6cycle	600	iv	Chemotherapy period	17
Shen X	2011	Chinese	30	18	ST-T segment change(9)QT-QTc change(2)QRS low voltage(3)	Sinus tachycardia(2)Atrial premature beat(3)Ventricular premature beat(1)	EPB	/	/	/	Chemotherapy period	16
Ma YF	2016	Chinese	40	10	R-wave low voltage(2)T wave change(7)ST segment change(4)QT interval change(1)	Sinus tachycardia/Sinus bradycardia(1)RBBB(2)PAC(1)PVC(1)AVB(2)	TE	1cycle	75	iv	Chemotherapy period	16
Ma YF	2016	Chinese	40	15	R-wave low voltage(6) T wave change(11) ST segment change(15) QT interval change(1)	Sinus tachycardia/Sinus bradycardia(4)RBBB(3)PAC(3)PVC(2)AVB(2)	TE	2cycle	150	iv	Chemotherapy period	16
Ma YF	2016	Chinese	40	21	R-wave low voltage(9) T wave change(14) ST segment change(20) QT interval change(5)	Sinus tachycardia/Sinus bradycardia(7)RBBB(5)PAC(7)PVC(5)AVB(3)	TE	3cycle	225	iv	Chemotherapy period	16
Qi YX	2017	Chinese	152	43	R-wave low voltage(29)T wave change(9)ST segment elevation(36)ST segment depression(19)QT interval change(23)	Atrioventricular block(9)RBBB(3)Sinus tachycardia(16)Sinus bradycardia(18)Premature beat(9)	AC-T	3cycle	225	/	Chemotherapy period	14
Wu QD	2016	Chinese	70	35	QRS low voltage(4)ST-T segment change(6)	Sinus bradycardia(4)Sinus tachycardia(8)Atrial premature contraction(4)Ventricular premature contraction(3)Supraventricular tachycardia(1)Bundle branch block(3)Atrioventricular block(2)	AC/FAC	6cycle	300–400	iv	Chemotherapy period	15
Wu QD	2016	Chinese	70	23	QRS low voltage(2)ST-T segment change(4)	Sinus bradycardia(3)Sinus tachycardia(5)Atrial premature contraction(3)Ventricular premature contraction(2)Bundle branch block(3)Atrioventricular block(1)	AC/FAC	6cycle	200–300	iv	Post-chemotherapy	15
Liu B	2018	English	409	101	ST-T segment change(45)QRS low voltage(14)	Sinus tachycardia(37)Sinus arrhythmia(8)Sinus bradycardia(4)Ventricular premature contraction(3)Atrial premature contraction(2)RBBB(2)Atrial flutter and borderline premature beat(1)	EC/CAF/EC-T/ET/TAC	4-6cycle	360–540、200–300	/	Chemotherapy period	15
Hu G.	2022	English	60	28	ST-T segment change(2)QT-QTc change(3)QRS low voltage(3)	Atrial premature beat(6)Ventricular premature beat(4)Sinus tachycardia(4)Sinus bradycardia(4)Ventricular block(2)	ADR	6cycle	300	iv	Chemotherapy period	16
Hu G.	2022	English	62	35	ST-T segment change(3)QT-QTc change(3)QRS low voltage(2)	Atrial premature beat(7)Ventricular premature beat(5)Supraventricular tachycardia(2)Sinus tachycardia(7)Sinus bradycardia(4)Ventricular block(2)	ADR	6cycle	450	iv	Chemotherapy period	16
Huang ZQ	2004	English	250	107	Limb lead low voltage(12)T wave change(29)T wave change(12)ST segment depression(18)QT interval change(28)	Sinus tachycardia(8)	AC/EC	10cycle	70	iv	Chemotherapy period	15
Ando M	2000	English	39	11	ST-T segment change(2)	Atrial arrhythmia(3)Ventricular arrhythmia(2)Atrioventricular block(4)	ADR	6-8cycle	300	iv	Chemotherapy period	13

### Results of the meta-analysis

#### Incidence of anthracycline-associated arrhythmias

**Fig 2:**
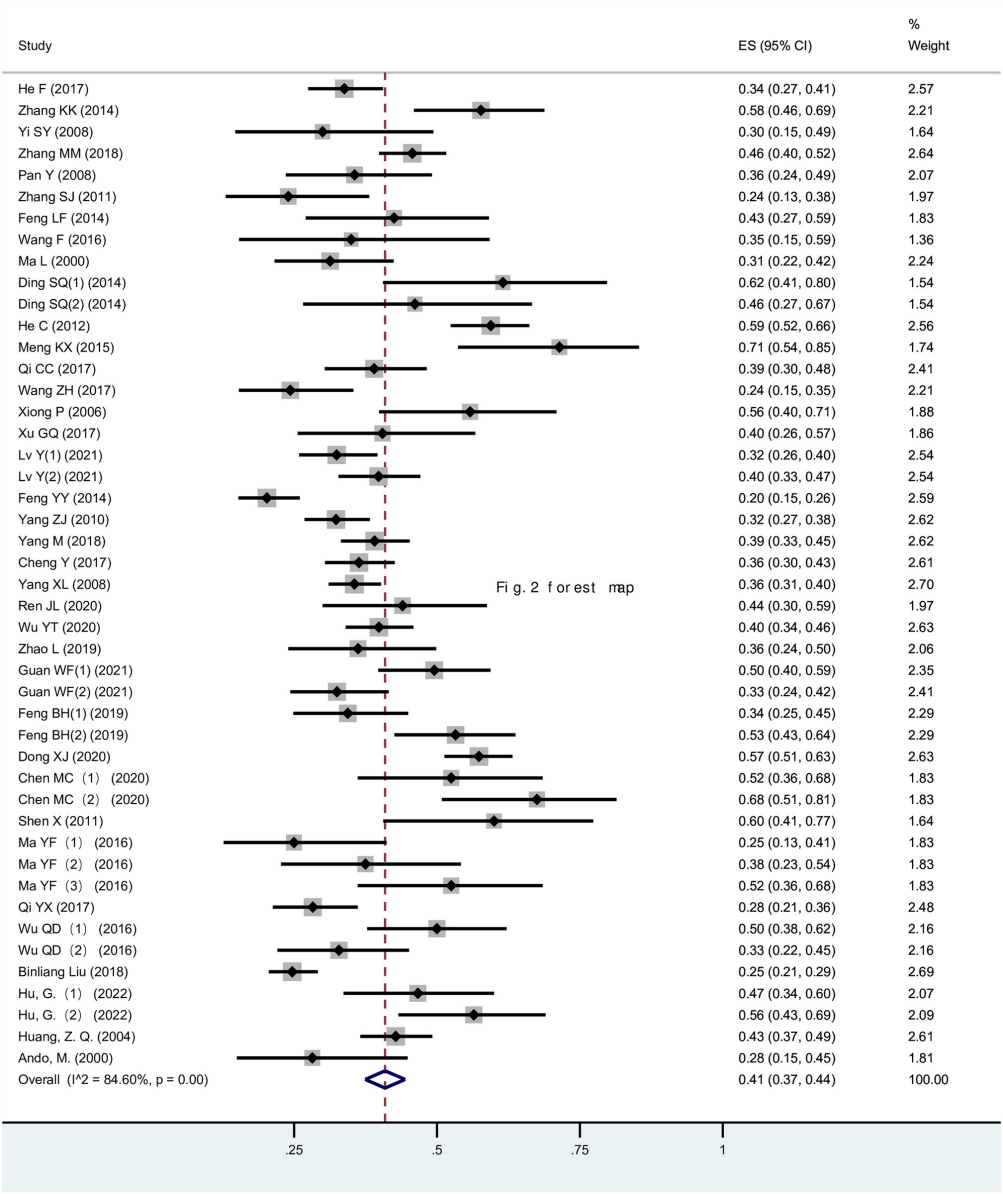
Forest plot of the incidences of anthracycline-induced arrhythmia and abnormal electrocardiogram changes in breast cancer.

A total of 2,257 anthracycline-associated arrhythmia events were reported across the 37 studies (n = 5705), with a wide incidence range from 20% to 71%. Due to significant heterogeneity between studies (I2 = 84.6%, P <0.001), a random effects model was employed for analysis. Fig 2 depicts a forest plot of the incidences of anthracycline-induced arrhythmias in breast cancer. A pooled analysis of the 37 studies revealed that the incidence of cardiac electrophysiological changes and arrhythmias in breast cancer patients receiving anthracycline chemotherapy was approximately 0.41 (0.37, 0.44).

#### Subgroup analysis

*Subgroup analysis based on publication year*, *sample size*, *dose*, *duration of administration and treatment plan and the timing of cardiac toxicity*. Subgroup analysis was conducted based on the year of publication, sample size, and dose and duration of anthracycline administration to explore the source of heterogeneity. As shown in Table 2, the treatment cycle may be one of the sources of heterogeneity in this study. The results (shown by year of publication in Table 2) indicated that the incidence of anthracycline-associated arrhythmias was 0.39 (0.32, 0.46) in 11 treatment groups of 11 studies published before 2014 and 0.41 (0.37, 0.46) in 35 treatment groups of 26 studies published in 2014 and after. The heterogeneity between groups was p = 0.604.

**Table 2:** Subgroup analysis based on publication year, sample size, and dose and duration of administration.

	Datasets	Total	Event	Proportion (95% CI)	*p*	*I* ^*2*^	heterogeneity between groups(P)
Year of publication							
Before 2014	11	1499	594	0.39(0.32,0.46)	<0.001	84.34%	0.604
After 2014	35	4206	1638	0.41(0.37,0.46)	<0.001	85.08%	
Sample size							
<100	28	1434	618	0.44(0.39,0.49)	<0.001	71.76%	0.158
100–200	6	884	322	0.37(0.31,0.42)	0.010	67.57%	
>200	12	3171	1220	0.39(0.32,0.45)	<0.001	94.32%	
Dosage							
<200mg/m^2^	3	330	132	/	/	/	0.304
200-399mg/m^2^	19	2342	928	0.41(0.35,0.47)	<0.001	87.25%	
≥400mg/m^2^	12	963	403	0.46(0.39,0.54)	<0.001	77.56%	
Cycle							
<4 cycle	5	463	151	0.34(0.27,0.41)	0.050	57.06%	0.040
4 cycle	8	955	457	0.50(0.41,0.58)	<0.001	82.63%	
6 cycle	18	1801	750	0.43(0.38,0.49)	<0.001	79.14%	
>6 cycle	6	760	315	0.39(0.31,0.47)	<0.001	74.55%	
Treatment plan							
monotherapy	13	1613	643	0.41(0.33,0.48)	<0.001	88.67%	0.766
combination therapy	33	4092	1614	0.42(0.38,0.46)	<0.001	85.59%	
The timing of cardiac toxicity							
chemotherapy period	42	5141	2156	0.42(0.38,0.46)	<0.001	86.70%	0.034
post-chemotherapy	4	291	101	0.41(0.38,0.45)	<0.001	0.00%	

For the analysis based on sample size (Table 2), subgroup analysis revealed that the incidence of anthracycline-associated arrhythmias was 0.44 (0.39, 0.49) in 27 treatment groups of 20 studies with small sample sizes(n<100). The incidence of anthracycline-associated arrhythmias was 0.37 (0.31, 0.42) in six treatment groups of five studies with moderate sample sizes(100<n<200). The incidence of anthracycline-associated arrhythmias was 0.39 (0.32, 0.45) in 12 treatment groups in 12 studies with large sample sizes(n>200). The intergroup heterogeneity was P = 0.158.

A subgroup analysis according to dose of anthracycline was also performed (Table 2). The results of the subgroup analysis showed that the number of studies in Group A(cumulative dose < 200 mg/m2) was less than or equal to 3, which was too low for STATA and Metaprop to be used for the subgroup analysis (the number of study groups needs to be > 3). Therefore, subgroup analysis could not be performed. There were 16 studies in Group B and 19 in the treatment group, and the combined analysis showed that the cumulative dose of anthracycline ranged from 200 mg/m2 to 399 mg/m2. The incidence of arrhythmia was 0.41 (0.35, 0.47). There were 10 studies in Group C and 12 in the treatment group. The combined analysis results showed that the cumulative dose of anthracycline was ≥400 mg/m2. The incidence of arrhythmia was 0.46 (0.39, 0.54). The intergroup heterogeneity was p = 0.304.

Subgroup analysis based on number of anthracycline cycles received was also performed (Table 2), subgroup analysis showed that group A (less than four cycles) included 3 studies and 5 treatment groups. Combined analysis showed that the incidence of arrhythmias was 0.34 (0.27, 0.41) with no more than 3 cycles of anthracycline therapy. A total of 8 studies and 8 treatment groups were included in Group B(four cycles), and the combined analysis showed that the incidence of arrhythmia was 0.50 (0.41, 0.58). A total of 14 studies and 18 treatment groups were included in Group C(six cycles), and the combined analysis results showed that the incidence of arrhythmia was 0.43 (0.38, 0.49). There were 6 studies and 6 treatment groups in Group D(more than six cycles), and the combined analysis showed that the incidence of arrhythmia was 0.39 (0.31, 0.47). The intergroup heterogeneity was P = 0.04 (< 0.05), so chemotherapy cycle may be one of the sources of heterogeneity in this study.

In addition, we conducted a subgroup analysis on different treatment plan. There were 13 groups that used anthracyclines alone and 33 groups that used anthracyclines in combination with other chemotherapy drugs. The results showed that the heterogeneity between these groups was 0.776, which is greater than 0.05. This result may suggest that there is no significant difference in the impact on arrhythmias, whether anthracyclines are used alone or in combination with other chemotherapy drugs. Moreover, the occurrence of cardiac toxicity at different times may contribute to the high heterogeneity(P = 0.034).

#### Subgroup analysis based on type of ECG change

Subgroup analysis was conducted based on the type of ECG change, which showed that the incidence of QT-QTc interval change was 0.08 (0.05, 0.11), that of P wave change was 0.10 (0.05, 0.15), that of ST-T segment change was 0.19 (0.15, 0.23), that of ST segment change was 0.17 (0.09, 0.27), and that of T segment change was 0.15 (0.12, 0.19). The incidence of low voltage abnormalities was 0.05 (0.03, 0.08), with an incidence of QRS low voltage being 0.04 (0.03, 0.06), and an incidence of R wave low voltage being 0.16 (0.09, 0.24). The details are presented in Table 3.

**Table 3:** Subgroup analysis based on arrhythmia.

	Datasets	Total	Event	Proportion (95%CI)	*p*	*I* ^*2*^
***ECG changes***						
**QT-QTc interval**						
QT-QTc interval change	20	2291	255	0.08 (0.05,0.11)	<0.001	85.40%
**P wave**						
P wave change	3	175	18	0.10(0.05,0.15)	0.530	0.00%
**ST-T segment**						
ST-T segment change	45	5144	1011	0.19(0.15,0.23)	<0.001	92.77%
ST segment change	9	1337	197	0.17(0.09,0.27)	<0.001	94.36%
T segment change	12	1880	289	0.15(0.12,0.19)	<0.001	76.20%
**Low voltage**						
Low voltage	24	2920	161	0.05(0.03,0.08)	<0.001	79.36%
QRS low voltage	15	1714	82	0.04(0.03,0.06)	<0.001	60.26%
R-wave low voltage	4	272	89	0.16(0.09,0.24)	0.060	58.73%
***Arrhythmia***						
**Conduction block**						
Conduction block	29	3859	127	0.04(0.02,0.05)	<0.001	76.47%
Branch bundle block	14	1798	47	0.03(0.02,0.04)	<0.001	59.20%
complete right bundle branch block	7	977	19	0.03(0.01,0.05)	<0.001	73.18%
Atrioventricular block	21	2724	57	0.02(0.01,0.03)	<0.001	61.47%
**Heart rate**						
Heart rate change	42	4977	576	0.12(0.10,0.15)	<0.001	82.95%
Tachycardia	30	4003	285	0.07(0.06,0.09)	<0.001	57.41%
Bradycardia	18	2866	130	0.05(0.03,0.06)	<0.001	66.21%
Supraventricular tachycardia	4	493	6	0.01(0.00,0.02)	0.310	16.23%
**Premature beat**						
Premature beat	38	4716	399	0.09(0.07,0.11)	<0.001	86.63%
Atrial premature beat	27	3487	179	0.05(0.04,0.07)	<0.001	80.20%
Ventricular premature beat	29	3972	163	0.04(0.02,0.05)	<0.001	74.41%
**atrial fibrillation**						
atrial fibrillation	3	546	17	0.04(0.00,0.12)	<0.001	89.67%

It is worth noting that Yang M’s study was the only one to report a short PR interval induced by anthracycline chemotherapy, with an incidence of 0.01. A study conducted by Chen MC explored the influence of treatment cycle on the incidence of short PR interval. The incidence was 0.55 with 4 cycles of chemotherapy and increased to 0.65 with 6 cycles, showing a certain dose-related relationship.

#### Subgroup analysis based on arrhythmia

Finally, subgroup analysis based on arrhythmia subtype was conducted. The results showed that the incidence of conduction block was 0.04 (0.02, 0.05), with the incidence of bundle branch block being 0.03 (0.02, 0.04), complete right bundle branch block being 0.03 (0.01, 0.05), and atrioventricular block being 0.02 (0.01, 0.03). The incidence of heart rate changes was 0.12 (0.10, 0.15), with the incidence of tachycardia being 0.07 (0.06, 0.09), bradycardia being 0.05 (0.03, 0.06), and atrioventricular block being 0.01 (0.00, 0.02). The incidence of premature beats was 0.09 (0.07, 0.11), with the incidence of atrial premature beats being 0.05 (0.04, 0.07) and ventricular premature beats being 0.04 (0.02, 0.05). The incidence of atrial fibrillation was 0.04 (0.00, 0.12). The details are presented in Table 3. Additionally, in the study by Chen MC et al. (n = 40), they observed 26 cases of short runs of ventricular tachycardia, 26 cases of frequent premature ventricular contractions, and 27 cases of paired premature ventricular contractions induced by anthracycline-based chemotherapy. On the other hand, Wu QD et al. reported one case of anthracycline-induced ventricular tachycardia in their study (n = 70). Furthermore, Cheng Y and Liu BL also reported a case of junctional premature beats.

*Other ECG changes*. Liu BL’s study (n = 409) reported a case of anthracycline-associated atrial flutter. Dong XJ’s study (n = 279) reported 3 patients with clockwise and 7 patients with counterclockwise flutter. Dong XJ’s study (n = 279) also reported 2 patients with Left ventricular hypertrophy.

### Publication bias

**Fig 3:**
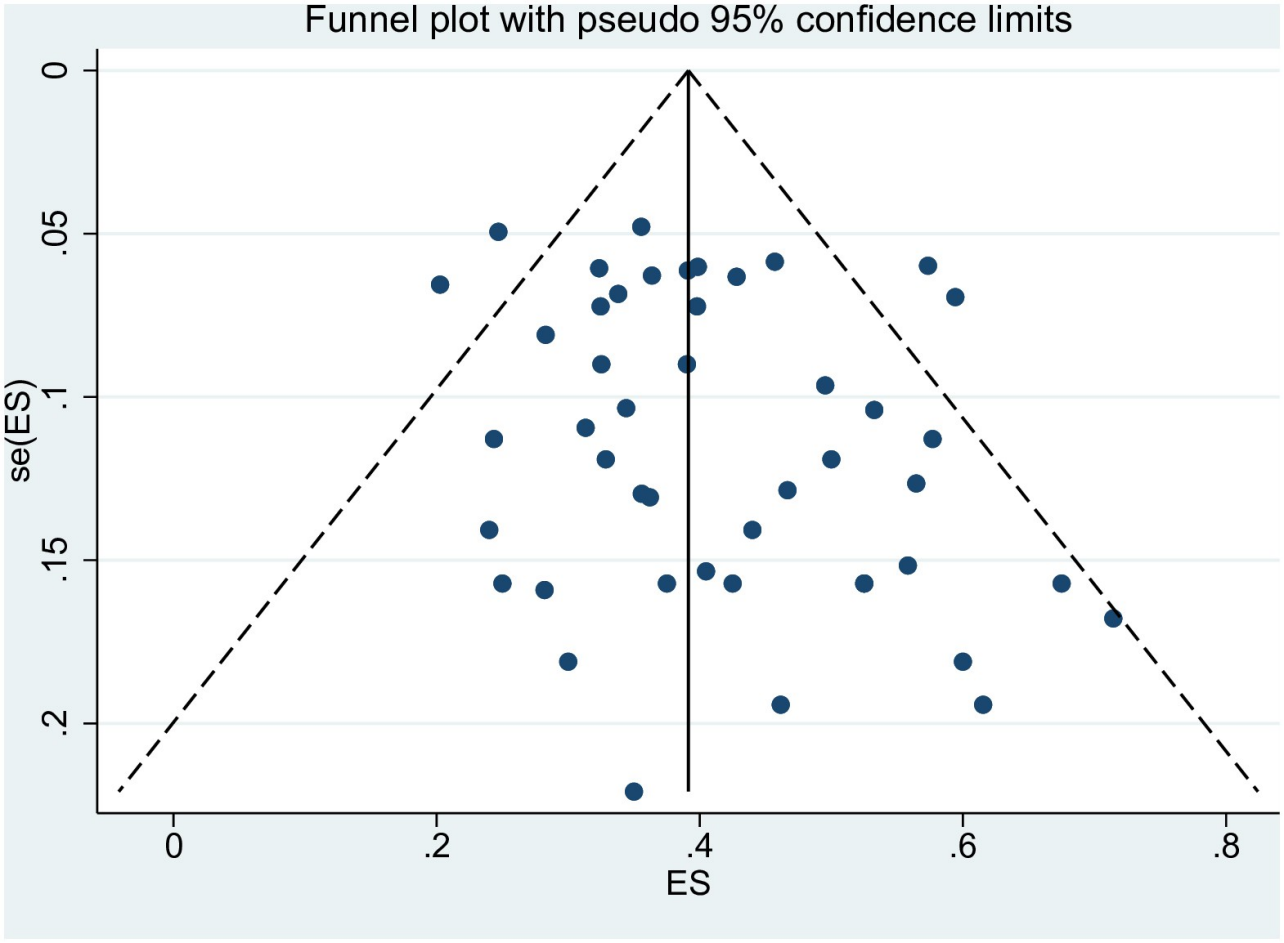
The funnel plot.

**Fig 4:**
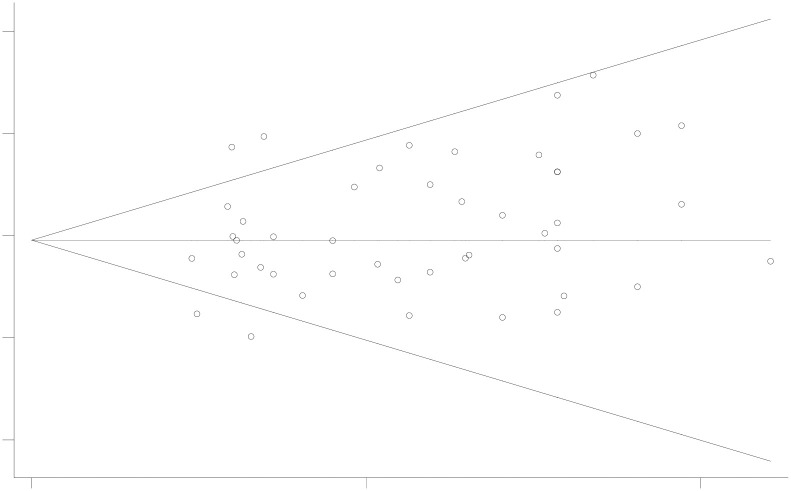
Begg’s test(P = 0.103).

**Fig 5:**
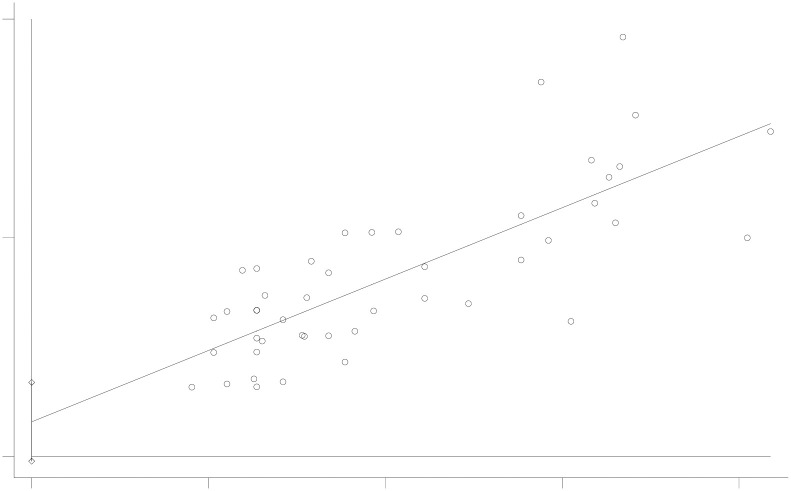
Egger’s test (P = 0.083).

Publication bias was evaluated using funnel plots and Begg’s and Egger’s tests. The funnel plot, depicted in Fig 3, shows that the included study scatter points were essentially symmetrically distributed along the midline. The completeness and symmetry of the funnel plot suggest no publication bias. Publication bias is defined as P<0.05 in Begg’s and Egger’s tests. The results of Begg’s test (P = 0.103) and Egger’s test (P = 0.083), shown in Figs 4 and 5, indicate no evidence of publication bias in this assessment of the incidence of anthracycline-related cardiotoxicity. Given the absence of significant publication bias in the included literature in this study, it was not necessary to evaluate the impact of publication bias on the results by sequentially removing each study and reanalyzing the data.

## Discussion

While anthracycline-based chemotherapy can enhance the quality of life and prolong the survival of breast cancer patients, a significant number of patients unfortunately develop various types of arrhythmias during or after anthracycline chemotherapy. These arrhythmias not only severely impact the patients’ quality of life but also pose a threat to their lives. Therefore, an accurate assessment of the incidence of anthracycline-related arrhythmias in breast cancer patients is crucial to guide the safe use of anthracyclines. To raise awareness of the risks associated with anthracycline-associated arrhythmias and to guide drug safety, we evaluated the incidence of arrhythmias and ECG abnormalities in breast cancer patients undergoing anthracycline chemotherapy.

We employed an extensive search strategy to capture all relevant literature and excluded studies that did not clearly report the details of anthracycline therapy to accurately assess the impact of each influencing factor on the incidence. Although we had to exclude approximately half of the articles from the meta-analysis due to a lack of details about the treatment regimen, the findings of the excluded articles were generally consistent with the pooled quantitative results, and there was no evidence of publication bias. Ultimately, a meta-analysis of 37 studies addressing the specific research questions was conducted. We found that the incidence of cardiac electrophysiological changes and arrhythmias was approximately 41% among breast cancer patients receiving chemotherapy with anthracyclines, and subgroup analysis showed that the treatment cycle and the timing of cardiac toxicity was a factor affecting incidence. Yang XL’s study described the detection rate of anthracycline-associated arrhythmias of both 12-lead electrocardiogram and dynamic electrocardiogram, and the results showed that the detection rate of dynamic electrocardiogram (0.41, 182/436) was higher than that of 12-lead electrocardiogram (0.36, 155/436) [16].

This study’s analysis of cardiac electrophysiological changes and arrhythmias found that anthracycline-based chemotherapy drugs could induce a variety of ECG changes, including alterations in the QT, PR interval period, ST segment, T wave, and ST-T changes, abnormal Q wave, abnormal QRS wave, conduction abnormalities (including conduction block, complete right bundle branch block, incomplete right bundle branch block, bundle branch block, left anterior branch block, intraventricular conduction block, and 1st to 3th degree atrioventricular block), heart rate changes (including arrhythmia, tachycardia, bradycardia, ventricular tachycardia, short array ventricular tachycardia and ventricular tachycardia), premature beat (including borderline premature beat, premature ventricular contraction, ventricular premature beat, frequent ventricular premature beat, and ventricular premature beat), low voltage abnormalities (including chest lead low voltage, limb and/or body lead low voltage, R wave low voltage, and QRS low voltage), axis deviation, clockwise rotation, counterclockwise rotation, among which ST-T segment change was the most common variation. Any changes on the electrocardiogram have their significance, such as changes in heart rate, and changes in the amplitude and duration of the electrocardiogram.” Studies have indicated that drug administration should be halted immediately when the QRS wave voltage decreases by 1/3 compared to the original voltage; otherwise, irreversible myocardial damage can occur, leading to heart failure[17]. However, QT prolongation is associated with syncope and sudden death as well as VT (Ventricular Tachycardia) and other rapid ventricular arrhythmias. An important ECG manifestation of anthracycline-induced ventricular arrhythmias is QT prolongation and the risk of progression to ventricular arrhythmias [18]. In the future, the potential of electrocardiograms will be further explored.

Regarding the pathogenesis of anthracycline-associated arrhythmias, Carmine Rocca et al. proposed that excessive reactive oxygen species (ROS) generation during anthracycline drug metabolism can inhibit the function of cardiac antioxidant enzymes, such as mitochondrial enzymes and NADPH oxidase. This results in damage to DNA, RNA, protein, and lipid molecular membranes, and typical redox modifications of macromolecules, including nitrotyrosine formation, lipid peroxidation, and protein carbonylation. In addition to the damage to cardiomyocytes themselves, oxidative stress also targets ion channels and affects ion membrane currents. This results in abnormal action potential propagation and arrhythmia [19]. Nathan H Waldron et al. pointed out that anthracyclines lead to arrhythmias by affecting autonomic nervous function, and that autonomic nervous imbalance caused by increased sympathetic activity or decreased vagal activity may cause ventricular arrhythmias. This mechanism may involve shortening the effective refractory period of the myocardium, shortening the duration of the action potential, increasing the dispersion of the effective refractory period, increasing the heterogeneity of repolarization, and inducing the early and later depolarization state. These electrophysiological changes reduce the threshold of ventricular arrhythmias and may induce ventricular arrhythmias [20]. Christos Kontogiannis et al. found that anthracyclines selectively bind to endogenous cardiolipin in mitochondria, resulting in mitochondrial accumulation and disruption of the electron transport chain via inhibition of mitochondrial complexes I and II; these effects result in additional ROS production and cardiolipin peroxidation. Mitochondrial damage can mediate the increase in calcium load and ROS production induced by adrenaline signaling and can induce the phosphorylation of the ryanodine receptor, leading to sarcoplasmic reticulum Ca2+ leakage, which leads to arrhythmia [21]. Additionally, anthracycline-based drugs can be mediated by reactive oxygen species groups, and free-state iron complex formation, and in the process, iron can generate ROS. Mitochondrial damage in cardiomyocytes and iron metabolism disorders can interact, leading to an extension of the action potential schedule, which increases the instability of the cell membrane potential and increases the probability of arrhythmia [22]. Thavendiranathan P et al. showed that anthracyclines can directly inhibit the adenylate-activated protein kinase signaling pathway and neuromodulin/ErbB signaling pathway in myocardial tissue, deplete the cardiomyocyte transcription factor GATA-4 and cardiac anchor chain repeat protein, induce activation and release of a series of inflammatory factors, inhibit transient and delayed outward potassium currents, and affect Na±Ca2+ exchange, leading to myocardial ischemia and hypoxia and bundle branch conduction system block and ultimately arrhythmia [23,24]. Shao WW et al. believed that the occurrence of arrhythmia may be related to calcium overload, increased cell membrane permeability, increased internal Ca2+ flow, inhibited Na±K±ATPase activity, decreased Na±K+ exchange, increased Na±Ca2+ exchange, accelerated internal Ca2+ flow, and activation of sarcoplasmic reticulum Ca2+ channels, increasing Ca2+ release [25]. Other related theories include apoptosis theory and theories related to energy metabolism disorders [26].

Cumulative drug dosage, age, gender, dietary habits, genetic predisposition, and medical history are all considered risk factors for anthracycline-induced cardiotoxicity [25]. Other studies have indicated that the concurrent use of anthracyclines with cyclophosphamide can enhance the cardiotoxicity of anthracyclines, although the effect is not significant when a conventional dose is used. This finding contradicts the report by Von Hoff et al., necessitating further research to elucidate the precise relationships. Current research suggests that the combination of anthracycline chemotherapy with cyclophosphamide may influence the outcomes of our study [27,28].

In terms of anthracycline-associated cardiotoxicity prophylaxis, Dexrazoxane (DZR) is known to prevent cardiac damage and is typically used with high-dose anthracyclines. However, its application in arrhythmia management remains to be studied [29]. Concurrently, dose control, slow intravenous infusion, and the use of anthracycline liposomes are considered effective strategies to reduce the incidence of cardiotoxicity [30]. Interestingly, physical exercise has also been found to confer a protective effect against anthracycline-related cardiotoxicity. Hornsby WE et al. discovered that aerobic training not only completely mitigated the detrimental effects of chemotherapy but also led to significant improvements in cardiopulmonary function during concurrent neoadjuvant therapy [31]. Studies have shown that traditional Chinese medicines such as ginkgo biloba extract, Wenxin granule, and Shenmai injection also exhibit protective effects against anthracycline-related cardiotoxicity [32,33].

There are several noteworthy limitations in the literature and our meta-analysis. Firstly, this study was not based on individual patient data, and the majority of the studies included in this meta-analysis were observational. Unlike randomized controlled trials, these observational studies may be more prone to inherent bias in research design, and those lacking a control group may be at risk of measurement bias. Secondly, due to flaws in study design, it was often unclear whether the arrhythmia events were attributable solely to anthracyclines, preexisting cardiovascular disease, other treatments (such as radiotherapy or other non-anthracycline agents like paclitaxel drugs), or confounding factors. In this paper, only studies with complete original data records were integrated and analyzed, and risk factors and other information evaluated based on subjective judgment and related epidemiological data were not integrated and systematically analyzed, so the data and analysis were relatively limited. Thirdly, although it is difficult for any meta-analysis to completely exclude the possibility of publication bias and we did not detect evidence of publication bias, we cannot completely exclude the possibility of publication bias in this study because we found that the incidence of arrhythmias was higher in the studies with smaller sample sizes. In addition, most of the studies included in this study were Chinese studies, and thus, regional differences may have affected the results. Fourthly, the present meta-analysis produced inconsistent incidence rates, and the software used for analysis varied. The specific operation steps and analysis methods, such as the publication bias analysis method and the method used to analyze differences, are not perfect, and the scarcity of literature may have affected the results. This meta-analysis provides strong supporting evidence that can lead to rapid improvements. Lastly, the literature covered in this study spans 22 years, from 2000 to 2022, during which significant advances have been made in the treatment of breast cancer and the detection of arrhythmias. However, our analysis found that earlier studies had similar incidence rates to newer studies.

## Conclusion

This study represents the first accurate assessment of the overall incidence of arrhythmias in breast cancer patients undergoing anthracycline treatment, as determined through meta-analysis. This provides crucial data support for clinical drug use and drug monitoring. The findings of this study assist physicians in better understanding and managing the potential cardiac toxicity that may be induced by anthracycline drugs in the treatment of breast cancer. Moreover, this article lays a significant foundation for future research, including further exploration of the specific mechanisms underlying anthracycline-induced arrhythmias, strategies for the prevention and treatment of this side effect, and investigations into whether other types of chemotherapy drugs also exhibit similar cardiotoxicity.

## Supporting information

S1 Table Literature search strate. (DOCX)

S2 Table PRISMA 2020 checklist. (DOCX)
