# Supplementary material for: Anthracycline-induced arrhythmias in breast cancer therapy: A meta-analysis of single-arm trials
Source: PLoS One. 2024 May 23;19(5):e0303208. doi: 10.1371/journal.pone.0303208 (PMC11115293; doi:10.1371/journal.pone.0303208)
Supplement: S1 Table — (DOCX) [file pone.0303208.s001.docx]

| Database | Keywords | N |
| --- | --- | --- |
| Web of Science | (Breast Neoplasm OR Breast Tumor OR Breast Cancer OR Mammary Cancer OR Malignant Neoplasm of Breast OR Breast Malignant Neoplasm OR Malignant Tumor of Breast OR Breast Malignant Tumor OR Cancer of the Breast OR Human Mammary Carcinoma OR Human Mammary Neoplasm OR Breast Carcinoma)AND (Aclarubicin OR Anthracyclines OR Daunorubicin OR Carubicin OR Doxorubicin OR Idarubicin OR Nogalamycin OR Plicamycin OR Adriamycin)AND (Cardiotoxicities OR Cardiac Toxicity OR Cardiac Dysrhythmia OR Cardiac Arrhythmia OR Cardiac Arrhythmias OR Arrhythmia OR Arrythmia) | 7855 |
| Embase | Breast Neoplasm OR Breast Tumor OR Breast Cancer OR Mammary Cancer OR Malignant Neoplasm of Breast OR Breast Malignant Neoplasm OR Malignant Tumor of Breast OR Breast Malignant Tumor OR Cancer of the Breast OR Human Mammary Carcinoma OR Human Mammary Neoplasm OR Breast Carcinoma in Keyword AND Aclarubicin OR Daunorubicin OR Carubicin OR Doxorubicin OR Idarubicin OR Nogalamycin OR Plicamycin OR Adriamycin in Keyword AND Drug Related Side Effects OR Adverse Reaction OR Drug Side Effect OR Adverse Drug Reactions OR Adverse Drug Event OR Side Effects of Drugs OR Drug Toxicity OR Cardiotoxicities OR Cardiac Toxicity OR Cardiac Dysrhythmia OR Cardiac Arrhythmia OR Cardiac Arrhythmias OR Arrhythmia OR Arrythmia in Keyword - in Trials (Word variations have been searched) | 300 |
| Cochrane Library | Breast Neoplasm OR Breast Tumor OR Breast Cancer OR Mammary Cancer OR Malignant Neoplasm of Breast OR Breast Malignant Neoplasm OR Malignant Tumor of Breast OR Breast Malignant Tumor OR Cancer of the Breast OR Human Mammary Carcinoma OR Human Mammary Neoplasm OR Breast Carcinoma in Keyword AND Aclarubicin OR Daunorubicin OR Carubicin OR Doxorubicin OR Idarubicin OR Nogalamycin OR Plicamycin OR Adriamycin in Keyword AND Drug Related Side Effects OR Adverse Reaction OR Drug Side Effect OR Adverse Drug Reactions OR Adverse Drug Event OR Side Effects of Drugs OR Drug Toxicity OR Cardiotoxicities OR Cardiac Toxicity OR Cardiac Dysrhythmia OR Cardiac Arrhythmia OR Cardiac Arrhythmias OR Arrhythmia OR Arrythmia in Keyword - (Word variations have been searched) | 324 |
| PubMed | ((Breast Neoplasm[Title/Abstract] OR Breast Tumor[Title/Abstract] OR Breast Cancer[Title/Abstract] OR Mammary Cancer[Title/Abstract] OR Malignant Neoplasm of Breast[Title/Abstract] OR Breast Malignant Neoplasm[Title/Abstract] OR Malignant Tumor of Breast[Title/Abstract] OR Breast Malignant Tumor[Title/Abstract] OR Cancer of the Breast[Title/Abstract] OR Human Mammary Carcinoma[Title/Abstract] OR Human Mammary Neoplasm[Title/Abstract] OR Breast Carcinoma[Title/Abstract]) AND (Aclarubicin[Title/Abstract] OR Daunorubicin[Title/Abstract] OR Carubicin[Title/Abstract] OR Doxorubicin[Title/Abstract] OR Idarubicin[Title/Abstract] OR Nogalamycin[Title/Abstract] OR Plicamycin[Title/Abstract] OR Adriamycin[Title/Abstract])) AND (((Drug Related Side Effects[Title/Abstract] OR Adverse Reaction[Title/Abstract] OR Drug Side Effect[Title/Abstract] OR Adverse Drug Reactions[Title/Abstract] OR Adverse Drug Event[Title/Abstract] OR Side Effects of Drugs[Title/Abstract] OR Drug Toxicity[Title/Abstract]) OR (Cardiotoxicities[Title/Abstract] OR Cardiac Toxicity[Title/Abstract])) OR (Cardiac Dysrhythmia[Title/Abstract] OR Cardiac Arrhythmia[Title/Abstract] OR Cardiac Arrhythmias[Title/Abstract] OR Arrhythmia[Title/Abstract] OR Arrythmia[Title/Abstract])) AND (clinicaltrial[Filter] OR randomizedcontrolledtrial[Filter]) Filters: Clinical Trial, Randomized Controlled Trial  (("breast neoplasm"[Title/Abstract] OR "breast tumor"[Title/Abstract] OR "breast cancer"[Title/Abstract] OR "mammary cancer"[Title/Abstract] OR "malignant neoplasm of breast"[Title/Abstract] OR "breast malignant neoplasm"[Title/Abstract] OR "malignant tumor of breast"[Title/Abstract] OR "breast malignant tumor"[Title/Abstract] OR "cancer of the breast"[Title/Abstract] OR "human mammary carcinoma"[Title/Abstract] OR (("human s"[All Fields] OR "humans"[MeSH Terms] OR "humans"[All Fields] OR "Human"[All Fields]) AND "mammary neoplasm"[Title/Abstract]) OR "breast carcinoma"[Title/Abstract]) AND ("Aclarubicin"[Title/Abstract] OR "Daunorubicin"[Title/Abstract] OR "Carubicin"[Title/Abstract] OR "Doxorubicin"[Title/Abstract] OR "Idarubicin"[Title/Abstract] OR "Nogalamycin"[Title/Abstract] OR "Plicamycin"[Title/Abstract] OR "Adriamycin"[Title/Abstract]) AND ("drug related side effects"[Title/Abstract] OR "adverse reaction"[Title/Abstract] OR "drug side effect"[Title/Abstract] OR "adverse drug reactions"[Title/Abstract] OR "adverse drug event"[Title/Abstract] OR "side effects of drugs"[Title/Abstract] OR "drug toxicity"[Title/Abstract] OR ("Cardiotoxicities"[Title/Abstract] OR "cardiac toxicity"[Title/Abstract]) OR ("cardiac dysrhythmia"[Title/Abstract] OR "cardiac arrhythmia"[Title/Abstract] OR "cardiac arrhythmias"[Title/Abstract] OR "Arrhythmia"[Title/Abstract] OR "Arrythmia"[Title/Abstract])) | 328 |
| Chinese National Knowledge Infrastructure (CNKI) | [(主题=乳腺癌 + 乳岩 + 乳癌 + 乳腺肿瘤) AND (主题=蒽环类 + 多柔比星 + 表柔比星 + 吡柔比星 + 柔红霉素 + 阿柔比星 + 伊达比星 + 安柔比星) AND (主题=安全性 + 不良反应 + 心脏毒性 + 心律失常)](https://kns.cnki.net/KNS8/AdvSearch?id=388&dbcode=SCDB&searchtype=gradeSearch&ishistory=1" \t "_blank" \o "(主题=乳腺癌 + 乳岩 + 乳癌 + 乳腺肿瘤) AND (主题=蒽环类 + 多柔比星 + 表柔比星 + 吡柔比星 + 柔红霉素 + 阿柔比星 + 伊达比星 + 安柔比星) AND (主题=安全性 + 不良反应 + 心脏毒性 + 心律失常)) | 1369 |
| Wanfang Database | 题名或关键词:(乳腺癌 or 乳岩 or 乳癌 or 乳腺肿瘤) and 题名或关键词:(蒽环类 or 多柔比星 or 表柔比星 or 吡柔比星 or 柔红霉素 or 阿柔比星 or 伊达比星 or 安柔比星) and 题名或关键词:(安全性 or 不良反应 or 心脏毒性 or 心律失常) | 1019 |
| China Science and Technology Journal Database (VIP) | 关键词=乳腺癌+乳岩+乳癌+乳腺肿瘤 AND 关键词=蒽环类+多柔比星+表柔比星+吡柔比星+柔红霉素+阿柔比星+伊达比星+安柔比星 AND 关键词=安全性+不良反应+心脏毒性+心律失常 | 50 |
